# Interference with orco gene expression affects host recognition in *Diorhabda tarsalis*

**DOI:** 10.3389/fphys.2022.1069391

**Published:** 2022-12-20

**Authors:** Hong-Hao Chen, Youssef Dewer, Yan Wang, Shu-Qian Tan, Xiao-Li Liu, Wang-Peng Shi

**Affiliations:** ^1^ Department of Entomology and MOA Key Laboratory of Pest Monitoring and Green Management Chinese Medicinal Herbs Research Center and College of Plant Protection, China Agricultural University, Beijing, China; ^2^ Institute of Plant Protection, Ningxia Academy of Agriculture and Forestry Sciences, Yinchuan, China; ^3^ Phytotoxicity Research Department, Central Agricultural Pesticide Laboratory, Agricultural Research Center, Dokki, Egypt

**Keywords:** beetle, antenna transcriptome, odorant receptor, RNA interference, licorice

## Abstract

**Introduction:** *Diorhabda tarsalis* Weise is an important insect pest of the Chinese licorice *Glycyrrhiza uralensis* Fisch. Behavior of the beetle, including host location, oviposition site selection, self-defense, and aggregation, were regulated by plant volatiles or insect pheromones.

**Aim:** In this study, Identification of ORs and function research on orco were carried out, these could lead to the development of understand for olfaction mechanism in *D. tarsalis*.

**Methods:** ORs were identified by PacBio RS II platform to sequence the antennas of adult *D. tarsalis*, the function of orco was explored by dsRNA interference.

**Results:** 29 odorant receptor candidate genes of *D. tarsalis* were obtained, which code for 130–479 amino acids. Phylogenetic trees of olfactory receptors were constructed with 243 ORs from eight Coleoptera species. *DtarORco*, *DtarOR7* and *DtarOR26* are specifically expressed in the antenna, and the expression levels were significantly higher than other *Dtar*ORs in antenna, there were no differential expression between male and female beetles. An odorant coreceptor gene (*DtarORco*) has characteristics of an odorant receptor family member, the encoded mature protein has a predicted molecular weight of 53.898 kDa, dsRNA L4440 expression vectors were constructed and successfully transformed into ribonuclease III-deficient Escherichia coli strain HT115 DE3. After interference treatment, the relative expression level of *DtarORco* in *D. tarsalis* antennae significantly decreased and electrophysiological responses to host localization odor signals significantly decreased. At the same time, beetles lost the ability to locate hosts.

**Discussion:** The research on its mechanism of olfaction may lead to the development of new control measures that are environmentally friendly.

## 1 Introduction

*Diorhabda tarsalis* Weise feeds exclusively on Chinese licorice (*Glycyrrhiza uralensis* Fisch) a traditional medicine. *D. tarsalis* causes large economic losses yearly (Qi et al., 2008). Chemical pesticides are commonly used for population management, it is important to find alternative methods because of the side-effects of chemical insecticides. The olfactory system is vital to regulating insect behavior, such as host localization, mate-seeking, predator avoidance and selection of spawning sites, etc. Olfactory recognition is a complex biological process involving many protein molecules (Wicher 2015; Butterwick et al., 2018; Pelosi et al., 2018). Odorant receptors (ORs) are transmembrane proteins that are expressed on the membranes of olfactory neurons.

ORs can convert chemical signals to electrical signals. They contain seven transmembrane domains, with the N terminal located in the intramembrane region and the C terminal located at the extramembrane region. ORs are critical proteins for insect recognition of odor substances, insect ORs are associated with pheromones, volatile phytochemicals and other natural odor compounds (Wang et al., 2010). Olfactory neurons (ORNs) express the highly conserved non-classical receptor Orco and the classical receptor ORx. The resulting Orco-OR heterodimer controls the specificity of the ion channel and determines the specificity of odor molecules recognized by ORs (Sato et al., 2008). Research on *D. tarsalis* odorant receptors can establish the mechanism by which chemical signals are converted to electrical signals in the olfactory nerve and reveal the co-evolution process between the pest and its host. Odorant receptors can also provide information useful for the development of less toxic management techniques by RNAi and screening odorant ligands.

ORs in beetles from the Chrysomelinae such as *Pyrrhalta maculicollis*, *Pyrrhalta aenescens, Galeruca daurica*, *Diabrotica virgifera*, and *Ophraella communa* have been identified. The PacBio SMRT single molecule real-time sequencing technique has ultra-long reading capacity. It supports the mining of isoforms, homologs, superfamily genes and alleles and is a suitable method for OR identification (Gordon et al., 2015). Orco function of *Dendroctonus armandi, Tenebrio molitor, Ophraella communa, Rhodnius prolixus* and *Apolygus lucorum* have been explored by RNAi (Jackson et al., 2003; Zhou et al., 2014; Zheng et al., 2015; Zhang B. et al., 2016; Franco et al., 2016; Liu et al., 2016). It is necessary to identify all the ORs and confirm the function of critical genes in *D. tarsalis,* however, there have been no report on OR identification and Orco function.

Identification and function research on ORs could lead to the development of understand for olfaction mechanism in *D. tarsalis.* More importantly, it could help for implement comprehensive pest control measures. We tested the following hypotheses: 1) that ORs would be identified by PacBio RS II platform to sequence the antennas of adult *D. tarsalis*; and 2) that the function of orco would be explored by dsRNA interference.

## 2 Materials and methods

### 2.1 Insect housing

In 2020, *Diorhabda tarsalis* were obtained from the Chinese licorice field in Yinchuan city (106.2517 N, 38.27559 E), NingXia Hui Autonomous Region, China. The indoor growing conditions were: temperature: 25 ± 1°C, relative humidity: 60% ± 5% and a 14:10 h (L:D) photoperiod. Adult beetles and larvae in cages were fed Chinese licorice leaves collected from the field. Eggs and pupae were placed in culture dishes containing cotton balls for moisture retention and the culture dishes were placed in the dark for egg hatching and adult eclosion.

### 2.2 PacBio sequencing

A total of 500 pairs of male and female *D. tarsalis* were collected and combined respectively. Total RNA was extracted using an assay kit and Nanodrop was used to measure RNA purity and concentration. Agilent 2100 was used to measure RNA integrity and qualified samples had an OD (260/280) of 1.8–2.2, RIN ≥ 7.5, and nucleic acid electrophoresis was used to detect the integrity of RNA. Qualified samples were used for library construction. The SMARTer™ PCR cDNA Synthesis Kit (Clontech, Palo Alto, CA, USA) was used to synthesize full-length cDNA of mRNAs. PCR was used to amplify full-length cDNAs, and end-terminal repair was carried out on full-length cDNAs. Then, SMRT dumbbell adapters (SMRTbell Template Prep Kit) were ligated to cDNAs and exonuclease digestion was carried out to obtain sequencing libraries. After library construction was completed, Qubit2.0 was used for accurate quantitation and Agilent 2100 was used to measure the size of the libraries. After the library quality control was confirmed to meet expectations, PacBio RS II was used for full-length transcriptome sequencing, which was carried out by Biomarker Technologies Co. Ltd. (Beijing).

### 2.3 Transcriptome bioinformatics analysis

Transcriptome polymerase fragments were screened and sub-reads of length >50 bp and accuracy rate >0.80 were selected. Sequences were converted to reads of insert (RoI) based on the number of adapters. RoI sequences were classified as full-length non-chimeric sequence, full-length chimeric sequence, and non-full-length sequence based on the presence of the 3′ primer, 5′ primer, and polyA sequence. SMRT Analysis software Iterative Clustering for Error Correction (ICE) was used to cluster RoIs with similar sequences to obtain a consensus sequence. Non-full-length sequences were used to polish the consensus sequence to obtain high-quality sequences. CD-HIT (identity > 0.99) non-redundancy processing was used to obtain full-length transcripts of Pacbio transcriptome and BUSCO was used for integrity evaluation of the transcriptome (Sharon et al., 2013; Thomas et al., 2014; Gordon et al., 2015). BLAST software (ver. 2.2.26) was used for transcript functional annotation classification in the NR, Pfam, Swiss-Pro, KEGG, KOG, COG, eggNOG, and GO databases (Ashburner et al., 2000; Tatusov et al., 2000; Koonin et al., 2004; Li et al., 2013; Finn et al., 2014; The UniProt Consortium, 2017).

### 2.4 OR identification

We used ORF finder (https://www.ncbi.nlm.nih.gov/gorf/gorf.html) to predict open reading frames in transcripts with odorant receptor in the annotation information. Blastx (https://blast.ncbi.nlm.nih.gov/) was used to screen for candidate ORs that may be homologous to those in other insects (E < 10^–5^). HMMTOP (http://www.sacs.ucsf.edu/cgi-bin/hmmtop.py) was used to predict the transmembrane domains of the encoded protein, which were combined with protein sequence length, structural characteristics, Nr annotation results, phylogenetics, and expression spectrum characteristics to determine candidate Orco genes. PRALINE (http://www.ibi.vu.nl/programs/pralinewww) was used to display the aligned Orco sequences. NetPhos 2.0 Server (http://www.cbs.dtu.dk/services/NetPhos/) was used to predict protein phosphorylation sites and the ProtParam tool (http://web.expasy.org/protparam/) was used to analyze physicochemical characteristics. The 214 ORs (>200 aa) from eight Coleoptera species, and 29 *D. tarsalis* ORs were used to construct phylogenetic trees, of which 22, 19, 25, 34, 31, 27, 28, and 28 ORs were from *P. maculicollis*, *T. molitor*, *P. aenescens*, *Colaphellus bowringi*, *Leptinotarsa decemlineata*, *D. virgifera*, *Tribolium castaneum*, and *Anoplophora chinensis*, respectively. The ginsi parameter in MAFFT was used for alignment of phylogenetic trees and JTT + CAT model in FASTTREE was used for tree construction. iTOL v3 (https://itol.embl.de) was used to modify the phylogenetic trees.

### 2.5 RT-qPCR analysis

RT-qPCR was used to detect the expression levels of Orco and 15 ORs in various body sites in adult beetles, various developmental stages, and in the antennas afterRNAi treatment (Supplementary Table S1). Antennas, heads (without antennae), legs, thorax, abdomen, and elytra of male and female *D. tarsalis* adults, fresh eggs, 1st–3rd instar larvae, and pupae were collected. After 1, 3, 5, 7, and 9 d of Orco dsRNA interference, the antennae were collected from the dsRNA treatment group and the control group. Triplicates were collected for each sample that from 100 beetles. The tissue and cell extraction kit (TRIzol) kit were used to extract total RNA and PrimeScript™RT reagent Kit with gDNA Eraser (Perfect Real Time) was used to synthesize cDNA. The abm®EvaGreen qPCR MasterMix-no dye assay kit was used for fluorescence quantitation and Roche LightCycler480 was used for quantitative PCR. Tubulin was used as an internal reference for RT-PCT. Supplementary table 1 shows the gene amplification primers. Real-time PCR reaction program was: 95°C, 5 min of pre-denaturation, followed by 40 cycles of 94°C, 30 s for denaturation, and 60°C, 1 min of annealing and extension. The conditions for the melt curve were 95 °C for 10 s, 65°C for 5 s, and 95°C for 0.5 s.

### 2.6 Orco dsRNA interference

The nucleic acid fragments (300 bp) encoding the first and second transmembrane domains of *D. tarsalis* Orco (*DtarORco*) were used as interference targets and Ribobio (Guangzhou, China) was used to chemically synthesize the target fragments (Li et al., 2016). XhoI was used to linearize the L4440 vector and agarose gel electrophoresis was used to examine the linearization results. The target fragment and linearized plasmid were mixed in a 5:1 M ratio and ligated at 37°C for 30 min. The cloning sites were EcoRI and XhoI. After ligation, the plasmid was immediately transformed into ribonuclease III-deficient *Escherichia coli* strain HT115 DE3, incubation in 2 × YT broth medium containing 100 μg/ml ampicillin and 10 μg/ml tetracycline (Chen et al., 2016). The Single colony of HT115 transformant were cultured in LB at 37 °C overnight. The bacteria culture was diluted 100-fold in 2 × YT medium with above antibiotic, then incubated at 37°C to OD600 = 0.5. The bacteria were cultured for additional4 h at 37 °C after add 0.4 mM IPTG. The bacteria were collected by centrifugation at 5000 *g* for 10 min and resuspened in ddH_2_O to 250×. The suspension were sprayed on *G. uralensis* leaves. The same concentration of untransformed L4440 plasmid was used as a control. After *D. tarsalis* fed on *G. uralensis* leaves for 24 h, fresh leaves were provided.

### 2.7 Electrophysiology and behavioral measurements

Based on orco expression level, 3 days after OrcoRNAi treatment of *D. tarsalis*, electrophysiology and behavioral responses to hexanal (CAS: 66-25-1), Z-3-hexenal (CAS: 6789-80-6), and Z-3-hexenol (CAS: 928-96-1) were measured, with dichloromethane as a control. The measurement concentration was 10 μg/μL.

EAG measurement: Antennas were resected at the base from adult *D. tarsalis*. The incision end was connected to the reference electrode and the recording electrode was inserted at the tip of the antenna. A silver-silver chloride wire was inserted in the electrode and connected to a signal amplifier. The conducting solution was an aqueous solution of 750 mg/ml NaCl+35 mg/ml KCl+29 mg/ml CaCl_2_ 2H_2_O. 400 ml/min clean and moist air was continuously blown on the setup. 10 ul of test solution was added to 2.5 cm × 0.5 cm filter paper and allowed to equilibrate for 20 s inside a Pasteur pipette before stimulation was carried out. The stimulation duration was 0.2 s and stimulation interval were 2 min. EAG 2000 (Syntech, the Netherlands) was used to monitor the antenna signal and data was collected (Chen et al., 2019). Four replicates were measured for each treatment and five antennas were repeated each time.

Behavioral measurement: The Y-shaped olfactometer used ha a main arm length of 25 cm, two lateral arms of length 15 cm and a 45° angle between lateral arms. The internal diameter of the main and lateral arms was 3 cm. The two lateral arms were connected with 100 ml conical flask using a Teflon tube and the test compound and control solvent dichloromethane were added to the conical flask. Purified air was blown into the flasks at a flow rate of 0.5–0.6 L/min. During measurement, each insect was observed for 15 min and entrance into the lateral arm and retention for 1 min was used as the selection criteria. A total of 50 insects were measured for each treatment. After the end of the experiment for each group, all conical flasks and connecting tubes were thoroughly cleaned with alcohol and dried with a hair dryer.

### 2.8 Data analysis

The 2^**−(△△Ct)**^ method (Adnan et al., 2011; Livak and Schmittgen 2001) was used for RT-qPCR quantitative data processing. One-way ANOVA followed by Tukey’s multiple comparison was used for analysis of gene expression differences. Student’s *t* test was used for analysis of electrophysiological response differences between the two groups. χ^2^ was used to analyze behavioral differences. GraphPad Prism eight was used for the statistical analysis and graph plotting for all experimental data.

## 3 Results and analysis

### 3.1 PacBio full-length sequences

Full passes ≥0 and sequence accuracy >0.80 were used as criteria to obtain 632,484 reads of interest (ROI) from the original sequences. The mean length, number of ROI sequences, and passes of inserted sequences in the library were 2,398 bp, 0.95, and 14, respectively, and were used to evaluate the offline data (Supplementary Table S2).

A total of 448,083 FL non-chimeric fragments were obtained, accounting for 70.8% of all sequences. The mean length was 2203 bp. In addition, there were 101,895 no primer sequences (16.1%), 58,225 no poly-A sequences (9.2%), 14,575 short reads (2.3%), and 9,712 full-length chimeric sequences (1.5%) (Supplementary Table S3, Supplementary Figure S1A).

Iterative cluster analysis was carried out on ROI sequences to obtain 277,861 consensus transcripts, mean length was 2,364 bp, and accuracy was more than 99%. After polishing using non-full-length sequences, a total of 204,545 high-quality transcripts were obtained, accounting for 73.61% of all transcripts. CD-HIT non-redundancy analysis was carried out on high-quality transcripts and polished transcripts to obtain 63,493 transcript sequences. Integrity evaluation was carried out on the transcriptome after redundancy removal and a total of 1658 BUSCO groups were found. A total of 1345 intact BUSCO C) groups were found, accounting for 81.13%, including 793 single copy S) and 552 duplicated D) BUSCO groups. 66 fragmented BUSCO (F, 3.98%) and 247 missing BUSCO (M, 14.90%) groups were obtained (Supplementary Figure S1B), showing that the integrity of the transcriptome was reliable.

### 3.2 Functional annotation of non-redundant high-quality transcripts

Obtain annotation information for 52,436 transcripts (Supplementary Table S4), and annotation rate was 82.59%. Alignment results showed that the species with the highest homology in annotated transcripts obtained from the NR database (E-value≤1.0e^−5^) was *L. decemlineata,* which also belongs to the Chrysomeloidea superfamily followed by *Anoplophora glabripennis* (Coleoptera) and 27.9% and 25.9%, respectively of the transcripts were annotated (Supplementary Figure S2).

Transcripts with E-value <1.0e^−5^ for GO classification and 13,372 transcripts were classified into three major categories and 51 subcategories, 16,402 molecular functions, 16,363 cellular components, and 25,983 biological processes. Of the 17 cell components subclasses, cellular components (3,527 transcripts, 26.38%), cells (3,526 transcripts, 26.37%), organelles (2,384 transcripts, 17.83%), cell membrane (2,310 transcripts, 17.27%), membrane components (1,627 transcripts, 12.17%), and macromolecular complexes (1,544 transcripts, 11.55%) were annotated. Of the 14 molecular function subclasses, binding (7,135 transcripts, 53.36%) and catalytic activity (6,558 transcripts, 49.04%) were annotated. Of the 20 biological process subclasses, metabolic processes (7,872 transcripts, 58.87%), cell transformation (6,306 transcripts, 47.16%), and single organism process (4,589 transcripts, 34.32%) were annotated. The above 11 subclasses are representative subcategories in GO classification. A total of 18,521 transcripts were divided into 25 subcategories based on COG classification. Of these, the top three subcategories by number of transcripts were replication, recombination, and repair (1,470 transcripts, 7.94%), post-translational modification, protein turnover, and chaperone functions (1,380 transcripts, 7.45%), and amino acid metabolism and transport (1,334 transcripts, 7.20%). The eggNOG database was used for functional description and functional annotation of orthologous groups and 46,455 transcripts were classified into 25 subgroups. With the exception of 20,673 transcripts without predicted annotation, the top three transcripts by number were post-translational modification, protein turnover, and chaperone (3,069 transcripts, 6.61%), intracellular trafficking, secretion, and vesicle transport (2,153 transcripts, 4.63%), and transcription (1,693 transcripts, 3.64%). A total of 25 subclasses were obtained after alignment with the KOG database, including 3,0676 transcripts, of which signal transduction mechanisms (3657 transcripts, 11.92%), post-translational modification, protein turnover, and chaperone (2456 transcripts, 8.00%), and general function prediction only (6475 transcripts, 21.11%) were representative subclasses. The KEGG database was used to predict 268 pathways containing 19,189 transcripts.

### 3.3 Identification of odorant receptors in *Diorhabda tarsalis*

29 candidate *D. tarsalis* odorant receptor genes were identified. These genes were given temporary names based on phylogenetics by which classical odorant receptors were named “*DtarORx*” (x = 1–28) and the non-classical odorant receptor was named *DtarORco* (Table 1). The candidate ORs encode for 130–479 amino acids of which intact ORFs were present for eleven ORs, accounting for37.93%. Odorant receptors contain 1–7 transmembrane domains and 24 (85.71%) ORs have more than five transmembrane helices. Twenty ORs with E-values of more than 10^−25^ and similarity > 50% were identified, accounting for 68.97%. The identified 29 ORs were homologous to eight other insects, of which 9, 9, 4, 2, 1, 1, 1, and one homolog was found in *P. maculicollis*, *P. aenescens*, *C. bowringi*, *G. daurica*, *A. chinensis*, *D. virgifera*, *Sitophilus oryzae*, and *A. glabripennis*, respectively. *DtarORco* possess classical odorant receptor characteristics as its sequences includes a 1440 bp open reading frame (ORF) that encodes 479 amino acids and shows extremely high conservation compared with homologs in the Coleoptera. Its amino acid similarity with non-classical odorant receptors in *O. communa*, *D. virgifera*, *L. decemlineata*, *C. bowringi*, *T. castaneum*, and *A. chinensis*) was 94.99%, 86.22%, 92.69%, 78.71%, 77.45%, 85.59%, and 90.81%, respectively (Supplementary Figure S3). *DtarORco* contains seven transmembrane helices, with the N-terminal located at the intramembrane region and C-terminal located at the extramembrane region. The 1st, 3rd, and 4th transmembrane helices have high sequence heterogeneity and the other four transmembrane domains have extremely high sequence similarity. The predicted molecular weight of the mature *DtarORco* protein is 3.898 kDa and its molecular formula is C_2462_H_3796_N_624_O_677_S_29_. *DtarORco* has an isoelectric point of 7.94, contains 35 negatively charged residues (Asp + Glu), and 37 positively charged residues (Arg + Lys). The hydrophobicity index (GRAVY) is 0.264 and the lipophilicity index is 99.75 indicating that the protein is strongly lipophilic. The predicted *in vitro* half-life in mammalian reticulocytes is 30 h, instability index II) is 34.55, and the protein is stable. A total of 42 phosphorylation sites were predicted, showing that there is diverse phosphorylation regulation of the protein after translation.

**TABLE 1:** List of odorant receptor genes in *D. tarsalis* antennal transcriptome.

Name	Length (nt)	ORF (aa)	Status	Tmd	E-value	Identity (%)	Score	Blastx best-hit	Species	Accession
DtarORco	1910	479	Complete	7	0	94.99	935	odorant receptor coreceptor	*Ophraella communa*	QEE83332.1
DtarOR1	939	251	5′ lost	4	5E-41	35.45	154	odorant receptor	*Anoplophora chinensis*	AUF73043.1
DtarOR2	1513	396	5′ lost	7	6E-50	27.18	183	odorant receptor 32	*Colaphellus bowringi*	ALR72575.1
DtarOR3	1373	388	5′ lost	7	4E-92	40.36	292	odorant receptor 30a-like	*Diabrotica virgifera*	XP_028135353.1
DtarOR4	1360	375	Complete	7	4.2E-26	31.76	113	odorant receptor 20	*Colaphellus bowringi*	ALR72565.1
DtarOR5	1358	401	5′ lost	7	2E-54	31	196	odorant receptor 49b-like	*Sitophilus oryzae*	XP_030759997.1
DtarOR6	1207	380	Complete	7	3E-133	50	396	odorant receptor 14, partial	*Pyrrhalta aenescens*	APC94327.1
DtarOR7	1244	390	Complete	7	0	68.21	587	odorant receptor 6, partial	*Pyrrhalta maculicollis*	APC94231.1
DtarOR8	1711	181	3′, 5′ lost	4	1E-33	67.02	140	odorant receptor 6, partial	*Pyrrhalta maculicollis*	APC94231.1
DtarOR9	1266	387	Complete	7	5E-134	50.65	399	odorant receptor 35	*Colaphellus bowringi*	ALR72578.1
DtarOR10	3218	130	3′, 5′ lost	1	6E-45	80	160	odorant receptor 22	*Pyrrhalta maculicollis*	APC94232.1
DtarOR11	1601	455	Complete	7	3E-103	39.54	324	odorant receptor 34	*Colaphellus bowringi*	ALR72577.1
DtarOR12	4590	217	5′ lost	3	1.78E-25	43	118	odorant receptor 25	*Pyrrhalta aenescens*	APC94326.1
DtarOR13	1373	414	5′ lost	5	0	89.8	763	odorant receptor 18	*Pyrrhalta maculicollis*	APC94230.1
DtarOR14	2807	393	Complete	7	0	65.99	555	odorant receptor 2	*Pyrrhalta aenescens*	APC94306.1
DtarOR15	1330	416	5′ lost	7	7E-88	50.77	277	odorant receptor	*Galeruca daurica*	QDD67757.1
DtarOR16	1071	325	3′, 5′ lost	6	2E-94	82.84	291	odorant receptor, partial	*Galeruca daurica*	QDD67757.1
DtarOR17	1152	363	3′, 5′ lost	7	0	81.06	516	odorant receptor 18	*Pyrrhalta aenescens*	APC94311.1
DtarOR18	1347	416	5′ lost	7	0	79.62	693	odorant receptor 26, partial	*Pyrrhalta aenescens*	APC94330.1
DtarOR19	1207	402	3′, 5′ lost	7	0	70.3	587	odorant receptor 5	*Pyrrhalta maculicollis*	APC94229.1
DtarOR20	1341	433	5′ lost	7	0	81	720	odorant receptor 5	*Pyrrhalta maculicollis*	APC94229.1
DtarOR21	2734	375	Complete	7	0	75.81	587	odorant receptor 11, partial	*Pyrrhalta maculicollis*	APC94238.1
DtarOR22	1286	378	Complete	7	1.6E-179	64.02	516	odorant receptor 12	*Pyrrhalta maculicollis*	APC94239.1
DtarOR23	2011	386	3′, 5′ lost	7	0	67.21	519	odorant receptor 8, partial	*Pyrrhalta aenescens*	APC94315.1
DtarOR24	1493	417	5′ lost	7	3E-129	50.37	392	odorant receptor Or1-like	*Anoplophora glabripennis*	XP_023310030.1
DtarOR25	1273	391	5′ lost	7	2E-123	45.88	372	odorant receptor 9, partial	*Pyrrhalta aenescens*	APC94236.1
DtarOR26	1324	401	Complete	7	0	82.7	670	odorant receptor 3, partial	Pyrrhalta aenescens	APC94308.1
DtarOR27	1296	388	Complete	5	1E-177	75.56	506	odorant receptor 1	Pyrrhalta maculicollis	APC94224.1
DtarOR28	1293	397	5′ lost	7	6E-169	58.66	488	odorant receptor 23, partial	Pyrrhalta aenescens	APC94324.1

### 3.4 Relative expression level of odorant receptors in *Diorhabda tarsalis*

**FIGURE 1:**
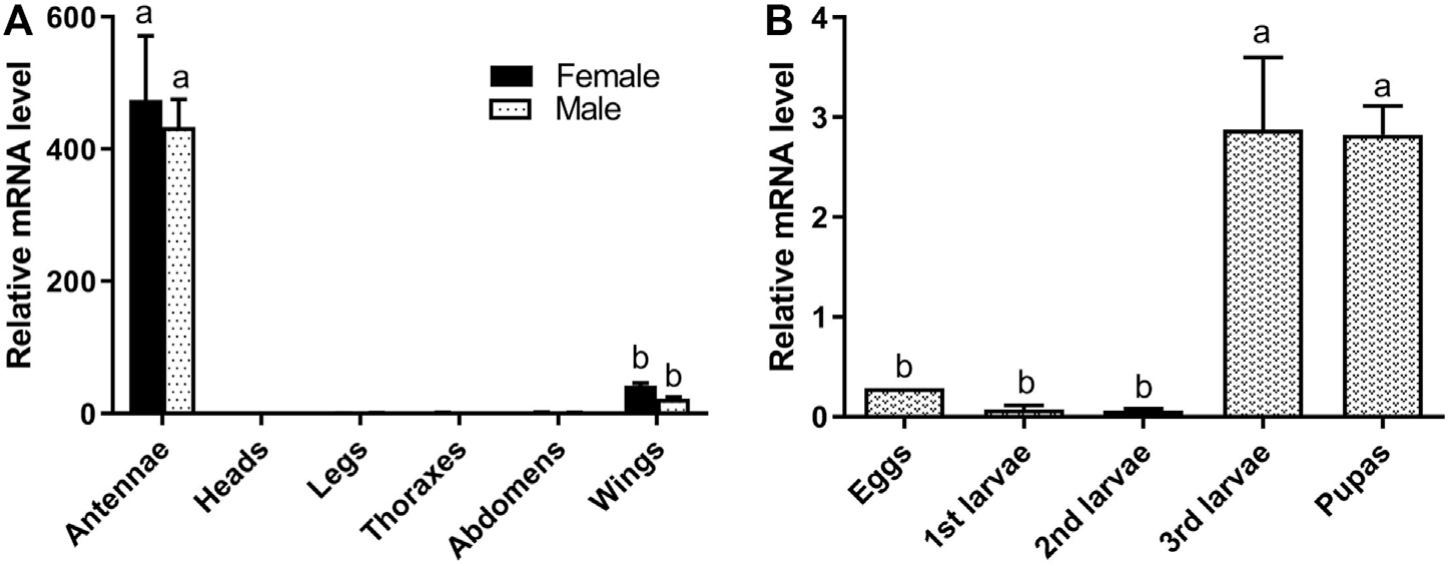
Relative expression levels of *DtarORco* in adult antenna, head, leg, thorax, abdomen, wing **(A)** and in different life stages or among different instars **(B)** using RTqPCR. The relative expression level is indicated as mean ± SE (N = 3). Different capital letters mean significant difference between tissues (*p* < 0.05).

RT-qPCR was used to measure the expression level of 15 randomly selected ORs and *DtarORco* in various organs of adult *D. tarsalis* and various life cycle stages of *D. tarsalis* (Figure 1, Supplementary Figures S4, 5). *DtarORco, DtarOR7* and *DtarOR26* shows extremely significant differences in relative expression level at various sites in adult beetles (F = 53.43, *p* < 0.01; F = 19.76, *p* < 0.01; F = 43.33, *p* < 0.01 respectively), and the expression levels were significantly higher than other *Dtar*ORs in antenna, *DtarORco* (female: 474.28 ± 137.60, male: 433.26 ± 73.50), *DtarOR7* (female: 31.91 ± 7.46, male: 23.70 ± 12.09) and *DtarOR26* (female: 60.39 ± 14.62, male: 78.50 ± 18.45) in antenna. Three genes were no differential expression between male and female beetles, and almost no expression in other sites except that *DtarORco* and *DtarOR7* had low expression in the elytra. In particular, the expression levels of *DtarOR12, DtarOR14, and DtarOR22* were high in the elytra. There were significant differences in relative expression level of most ORs (*DtarORco*) in different life stages and among different instars, however, the overall expression level was low, expression levels in pupae were higher than in the other life stages.

### 3.5 Phylogenetic analysis

**FIGURE 2:**
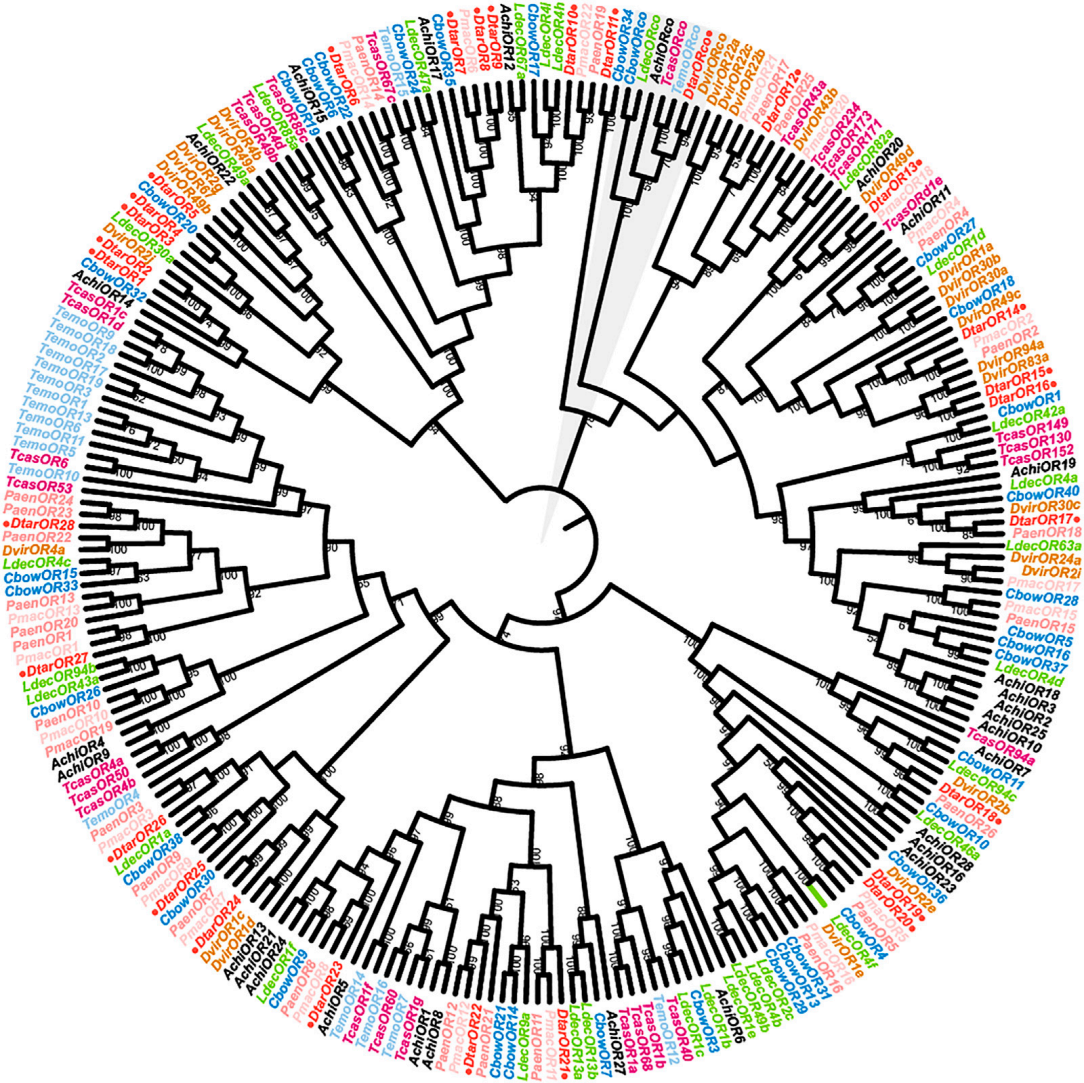
Phylogenetic tree of odorant receptor genes from *D. tarsalis* and other Coleoptera. Pmac, *Pyrrhalta maculicollis* (N = 22); Temo, *Tenebrio molitor* (N = 19); Paem, *Pyrrhalta aenescens* (N = 25); Cbow, *Colaphellus bowringi* (N = 34); Ldec, *Leptinotarsa decemlineata* (N = 31); Dvir, *Diabrotica virgifera* (N = 27); Tcas, *Tribolium castaneum* (N = 28); Achi, *Anoplophora chinensis* (N = 28).

A total of 29 *D. tarsalis* odorant receptors and 243 ORs from eight Coleoptera species (>200 aa) were used to construct phylogenetic trees (Figure 2). The overall support was high, showing that the phylogenetic relationship obtained was reliable. All OR sequences were clustered into seven branches and the 29 *DtarORs* were dispersed in various branches in the phylogenetic net. Orco from the species used for tree construction clustered together. *DtarOR1- DtarOR10, DtarOR12- DtarOR17, DtarOR18-DtarOR20, DtarOR21-DtarOR23*, and *DtarOR24-DtarOR28* clustered in the same branches while *DtarOR11* and *CbowOR34* from *C. bowringi* clustered in a single branch, showing that they have further phylogenetic distances compared with other odorant receptors. Most *DtarORs* and odorant receptors from Chrysomelinae species clustered together, showing that the homologous relationships and inter-species phylogenetic relationships of ORs were consistent.

### 3.6 Expression level changes after *DtarORco* RNAi

**FIGURE 3:**
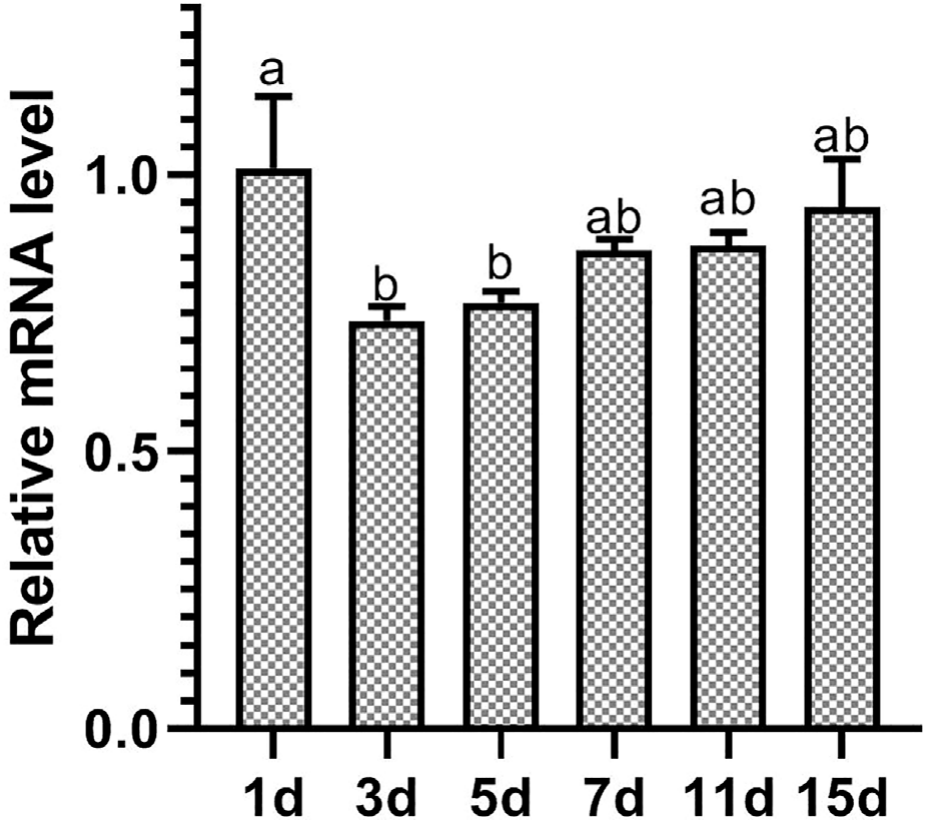
Expression levels of *DtarORco* after knockdown by RNAi (n = 3, *p* < 0.05).

After *D. tarsalis* fed on leaves that were sprayed with dsRNA-expressing HT115, RT-qPCR was used to measure the relative expression level of *DiarORco* on Days 1, 3, 5, 7, 11, and 15 (Figure 3). The differences in the relative expression level of *DtarORco* on different days after interference treatment were significant (F = 5.641, *p* = 0.0161). The differences between 1 d after interference and 3 d and 5 d after interference were significant, but other differences were not significant. The relative expression level on Day 1 was 1.01 ± 0.13, which was an increase compared with the control group that was fed untransformed L4440 plasmid. The Day 3 expression level was the lowest (0.74 ± 0.03) and was 26% lower than the control group. Subsequently, expression levels on various days gradually increased but were lower than the control group and the expression level on Day 15 was similar to the control group. This shows that the *DtarORco* expression level first increased before decreasing and then gradually increased after dsRNA interference in *D. tarsalis*. The decrease in the expression level was most significant on Day 3 of interference. The interference effects persisted for 15 d.

### 3.7 Electrophysiology and behavioral measurements after DtarORcoRNAi

**FIGURE 4:**
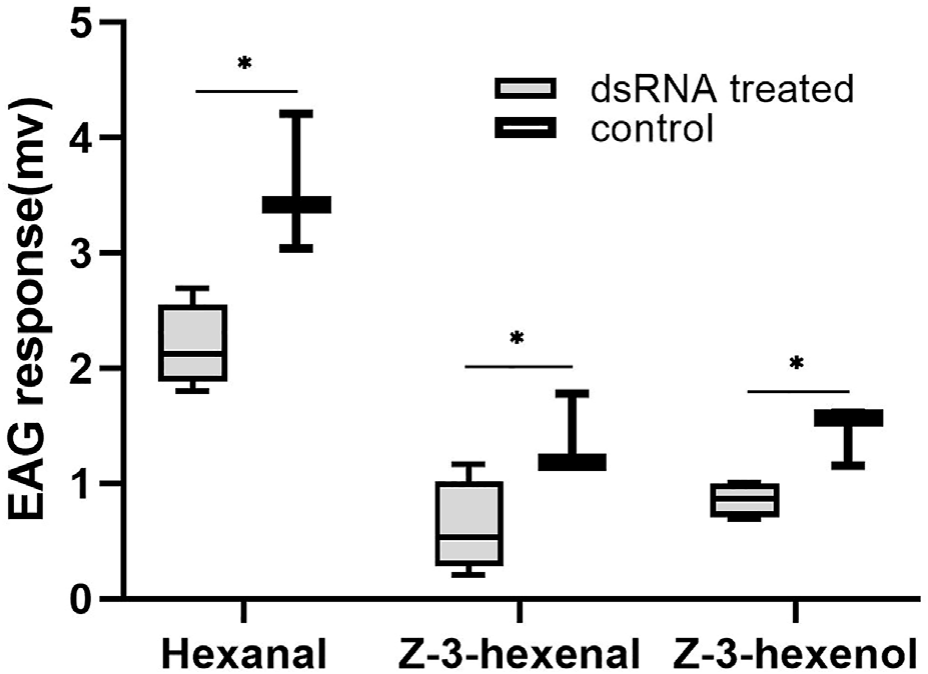
Electroantennogram (EAG) responses ofRNAi-treated *D. tarsalis* to host volatiles after 3 d *represents significant difference,**represents extremely significant difference (n = 4).

The EAG responses of *D. tarsalis* to three sensitive host volatile substances were measured 3 days after *DtarORco* dsRNA interference in *D. tarsalis* (Figure 4). After interference of *DtarORco* expression, antenna responses to the three volatile substances significantly decreased. The response time to hexanal was 2.19 ± 0.37 mv, which was a decrease of 38.47% compared to the control group and this difference was significant (t = 3.7794, *p* = 0.0129). The response time to Z-3-hexenal was 0.61 ± 0.40 mv, which was a decrease of 55.77% compared with the control group and this difference was significant (t = 2.6570, *p* = 0.0450). The response time to Z-3-hexenol was 0.86 ± 0.16 mv, which was a decrease of 40.60% compared with the control group and this difference was significant (t = 3.8146, *p* = 0.0124). Hexanal, Z-3-hexenal, Z-3-hexenol are signaling molecules that *D. tarsalis* uses to locate its host and decreased electrophysiological responses to these substances means that *DtarORco* interference decreases recognition of host odor molecules.

**FIGURE 5:**
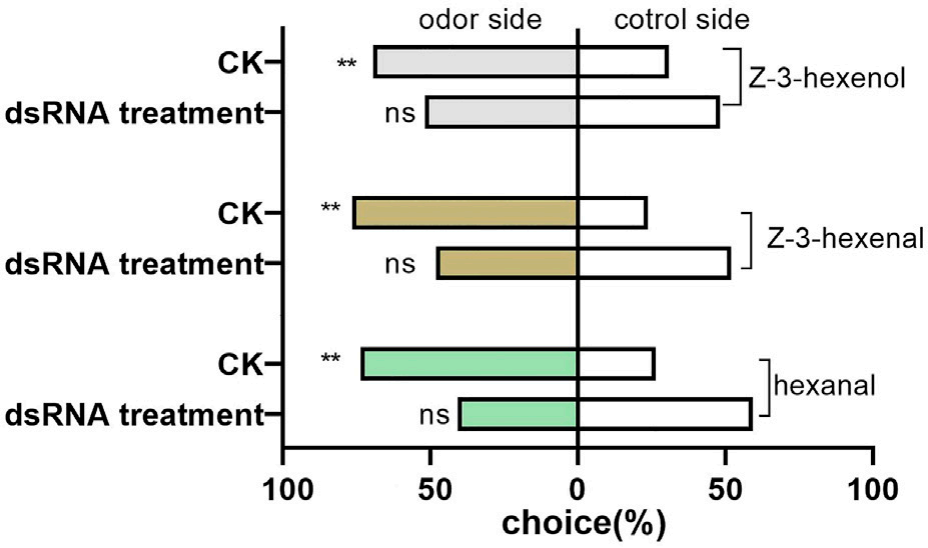
Behavioral response of *D. tarsalis* to three odorant signals for host location after *DtarORco* silencing. *represents significant difference,**represents extremely significant difference (n = 50).

The preferences of *D. tarsalis* to three sensitive host volatiles were not significant (hexanal: χ^2^ = 3.0695, *p* = 0.0798; Z-3-hexenal: χ^2^ = 0.0731, *p* = 0.7869; and Z-3-hexenol: χ^2^ = 0.0731, *p* = 0.7869), after *DtarORco* dsRNA interference 3 days, and they exhibited random selection behavior (Figure 5). In contrast, the control group exhibited extremely significant preference. The difference in selection success rate of *D. tarsalis* towards hexanal was extremely significant compared with the control group, which was a decrease of 44.72%. The difference in selection success rate of *D. tarsalis* towards Z-3-hexenal was significant compared to the control group, which was a decrease of 36.99%. The difference in selection success rate of *D. tarsalis* towards Z-3-hexenol was not significant compared with the control group, which was a decrease of 25.21%. These data show that host tracking signal and host localization capabilities were weakened in *D. tarsalis* after *DtarORco* interference.

## 4 Discussion

We obtained 29 *D. tarsalis* ORs and many full-length ORFs. This number is comparable to the number of ORs in other Coleoptera species, such as 26, 22, 21, 34, 24, 30, and 37 in *P. aenescens*, *P. maculicollis*, *G. daurica, Ambrostoma quadriimpressum*, *A. tetrariimpressum*, *O. communis*, and *L. decemlineata,* respectively, but lower than *C. bowringi*, *P. striolata*, and *B. longissima* that had 43, 73, and 48 ORs, respectively, and far fewer than *T. castaneum* (341 ORs) (Hunt et al., 2007; Mitchell et al., 2012; Li et al., 2015a; Zhang R. R. et al., 2016; Wang et al., 2016). This may be because the level of some transcripts is too low and not covered by transcriptome sequencing or the evolution of certain transcripts is too specific and cannot be annotated. Therefore, the identified odorant receptors cannot completely reflect the odorant receptors in the *D. tarsalis* antenna (Li et al., 2015b). However, the genome data of *D. tarsalis* has not been published and it is not possible to mine other OR genes. Quantitative analysis showed that 15 ORs are highly expressed in wings. For example, *DtarOR14* is specifically expressed in wings and its ecological function requires further study. In the phylogenetic trees constructed in this study, most *D. tarsalis* odorant receptor genes clustered with homologs in related species, while *DtarOR11* and *CbowOR34* from *C. bowringi* clustered together. This species-specific expansion was also found in the OR genes of *T. castaneum, M. caryae*, and *A. corpulenta*. Phylogenetic trees were constructed and *DtarORco* was found to have a complete ORF and typical characteristics of the odorant receptor family. In addition, *DtarORco* was highly expressed in the antenna. These findings increased understanding of the molecular mechanisms of olfactory recognition in *D. tarsalis*. The expression level of *DtarORco* significantly decreased whenRNAi targeting *DtarORco* was used. In addition, the ability to sense host volatile substances decreased and tactic behavior was lost. The study results provide a theoretical basis to better understand the molecular mechanisms of olfactory recognition in *D. tarsalis* and simultaneously proves the feasibility of green prevention and control using *D. tarsalisRNAi* against *DtarORco*.

Orco plays an important role in chemical olfactory recognition in insects (He et al., 2018) and knocking out this function can decrease their olfactory recognition capabilities (Soffan et al., 2016). For example, silencing Orco in *Ophraella communa* blocks its ability to seek hosts. This is because ORs cannot normally bind to Orco to form dimers and specifically transduce odor signals (Benton et al., 2006). Here *DtarORco* is expressed in the antennae of both male and female beetles and there was no statistical difference in expression level between the sexes. This is consistent with findings in *Drosophila melanogaster* (Menuz et al., 2014) and may be due to common ecological benefits in both males and females under normal circumstances. We found that *DtarORco* also has high expression in wings in addition to the antennae. *Sitobion avenae* uses the pheromone E-β-farnesene to induce wing bud development and olfactory recognition is associated with many biological processes (Fan et al., 2015). Similarly, *DtarORco* may have other unique functions in wings. In this study, dsRNA was used to silence *DtarORco* and there were significant changes in electrophysiology and behavioral phenotypes in *D. tarsalis* towards host volatile substances. This proved that *DtarORco* is indispensable to host seeking and localization by *D. tarsalis*. Therefore, regulation of host localization behavior using *DtarORco* as a target has application potential. Orco knockout not only silences host localization ability but also affects mating, reproduction, and population growth. Further research is needed to evaluate its combined effects. This study also conducted partial suitability research on the induction of *DtarORco* silencing. The C-terminal of Orco binds to the classical odorant receptor to form a dimer (Ma et al., 2020) and is highly conserved in insects. To design highly specific dsRNAs, the 1st and 2nd transmembrane domains in the N-terminal of *DtarORco* were used as targets to design a 300 bp dsRNA. The most direct effects of Orco silencing are significant changes in movement speed and movement paths. For example, Orco mutations in ants disrupted their foraging and induced a wandering phenotype (Yan et al., 2017). When *DtarORco* was silenced, the preferences of *D. tarsalis* towards three important host volatile substances were significantly decreased, demonstrating that Orco is vital for recognition of these three substances. The RT-qPCR results in this study showed that *DtarORco* expression was only partially silenced and the most significant reduction in relative expression level was only 26%. However, this decreased electrophysiological responses in antennae by 38.4%–55.77% and significantly decreased host localization behavior in *D. tarsalis*. This may have occurred because Orco needs to bind to many ORs and a small decrease in Orco significantly affected allocation to specific ORs. Although EAG response was somewhat decreased, it had reached the potential threshold for behavioral decisions.

## Data Availability

The deep sequencing dataset has been uploaded to the NCBI Short Read Archive database, BioProject ID number PRJNA312006. The Accession numbers are SRR3170921,SRR3170922 and SRR3170923.

## References

[B1] Adnan M. Morton G. Hadi S. (2011). Analysis of rpoS and bolA gene expression under various stressinduced environments in planktonic and biofilm phase using 2−ΔΔCT method. Mol. Cell. Biochem. 357, 275–282. 10.1007/s11010-011-0898-y 21630090

[B2] Ashburner M. Ball C. A. Blake J. A. Botstein D. Butler H. Cherry J. M. (2000). Gene ontology: Tool for the unification of biology. The gene ontology Consortium. Nat. Genet. 25, 25–29. 10.1038/75556 10802651 PMC3037419

[B3] Benton R. Sachse S. Michnick S. W. Vosshall L. B. (2006). Atypical membrane topology and heteromeric function of Drosophila odorant receptors *in vivo* . PLoS Biol. 4, e20. 10.1371/journal.pbio.0040020 16402857 PMC1334387

[B4] Butterwick J. A. del Mármol J. Kim K. H. Kahlson M. A. Rogow J. A. Walz T. (2018). Cryo-EM structure of the insect olfactory receptor Orco. Nature 560, 447–452. 10.1038/s41586-018-0420-8 30111839 PMC6129982

[B5] Chen W. Yu H. W. Ye L. D. (2016). Comparative study on different expression hosts for alkaline phytase engineered in *Escherichia coli* . Appl. Biochem. Biotechnol. 179, 997–1010. 10.1007/s12010-016-2046-3 26971961

[B6] Chen L. Li Y. Y. Shao K. M. (2019). A practical technique for electrophysiologically recording from lamellated antenna of scarab beetle. J. Chem. Ecol. 45, 392–401. 10.1007/s10886-019-01059-3 30825039

[B7] Fan J. Zhang Y. Francis F. Cheng D. Sun J. Chen J. (2015). Orco mediates olfactory behaviors and winged morph differentiation induced by alarm pheromone in the grain aphid, *Sitobion avenae* . Insect biochem. Mol. Biol. 64, 16–24. 10.1016/j.ibmb.2015.07.006 26187252

[B8] Finn R. D. Bateman A. Clements J. Coggill P. Eberhardt R. Y. Eddy S. R. (2014). Pfam: The protein families database. Nucleic Acids Res. 42, 222–230. 10.1093/nar/gkt1223 PMC3965110 24288371

[B9] Franco T. A. Oliveira D. S. Moreira M. F. Leal W. S. Melo A. C. A. (2016). Silencing the odorant receptor co-receptor RproOrco affects the physiology and behavior of the Chagas disease vector *Rhodnius prolixus* . Insect biochem. Mol. Biol. 69, 82–90. 10.1016/j.ibmb.2015.02.012 25747010

[B10] Gordon S. P. Tseng E. Salamov A. Zhang J. Meng X. D. Zhao Z. Y. (2015). Widespread polycistronic transcripts in fungi revealed by single- molecule mRNA sequencing. Plos One 10, e0132628. 10.1371/journal.pone.0132628 26177194 PMC4503453

[B11] He P. P. Engsontia P. Chen G. L. Yin Q. Wang J. Lu X. (2018). Molecular characterization and evolution of a chemosensory receptor gene family in three notorious rice planthoppers, *Nilaparvata lugens, Sogatella furcifera* and *Laodelphax striatellus*, based on genome and transcriptome analyses. Pest Manag. Sci. 74, 2156–2167. 10.1002/ps.4912 29542232

[B12] Hunt T. Bergsten J. Levkanicova Z. Papadopoulou A. John O. S. Wild R. (2007). A comprehensive phylogeny of beetles reveals the evolutionary origins of a superradiation. Science 318, 1913–1916. 10.1126/science.1146954 18096805

[B13] Jackson A. L. Bartz S. R. Schelter J. Kobayashi S. V. Burchard J. Mao M. (2003). Expression profiling reveals off-target gene regulation by RNAi. Nat. Biotechnol. 21, 635–637. 10.1038/nbt831 12754523

[B14] Koonin E. V. Fedorova N. D. Jackson J. D. Jacobs A. R. Krylov D. M. Makarova K. S. (2004). A comprehensive evolutionary classification of proteins encoded in complete eukaryotic genomes. Genome Biol. 5, R7. 10.1186/gb-2004-5-2-r7 14759257 PMC395751

[B15] Li W. Kondratowicz B. McWilliam H. Nauche S. Lopez R. (2013). The annotation-enriched non-redundant patent sequence databases. Database (Oxford) 2013, bat005. 10.1093/database/bat005 23396323 PMC3568390

[B16] Li X. Ju Q. Jie W. Li F. Jiang X. Hu J. (2015a). Chemosensory gene families in adult antennae of *Anomala corpulenta* motschulsky (Coleoptera: Scarabaeidae: Rutelinae). Plos One 10 (4), e0121504. 10.1371/journal.pone.0121504 25856077 PMC4391716

[B17] Li X. Zhu X. Y. Wang Z. Q. Wang Y. He P. Chen G. (2015b). Candidate chemosensory genes identified in *Colaphellus bowringi* by antennal transcriptome analysis. BMC Genomics 16, 1028. 10.1186/s12864-015-2236-3 26626891 PMC4667470

[B18] Li F. C. Gasser R. B. Lok J. B. Korhonen P. K. He L. Di W. D. (2016). Molecular characterization of the *Haemonchus contortus* phosphoinositide-dependent protein kinase-1 gene (Hc-pdk-1). Parasit. Vectors 9, 65. 10.1186/s13071-016-1351-6 26842781 PMC4741024

[B19] Liu X. M. Zhang B. X. Li S. G. Rao X. J. Wang D. M. Hu X. X. (2016). Knockdown of the olfactory co-receptor Orco impairs mate recognition in *Tenebrio molitor* (Coleoptera: Tenebrionidae). J. Asia. Pac. Entomol. 19, 503–508. 10.1016/j.aspen.2016.05.005

[B20] Livak K. J. Schmittgen T. D. (2001). Analysis of relative gene expression data using real-time quantitative PCR and the 2^− ΔΔCT^ method. Methods 25, 402–408. 10.1006/meth.2001.1262 11846609

[B21] Ma C. Cui S. W. Bai Q. Tian Z. Y. Zhang Y. Chen G. M. (2020). Olfactory co-receptor is involved in host recognition and oviposition in *Ophraella communa* (Coleoptera: Chrysomelidae). Insect Mol. Biol. 29, 381–390. 10.1111/imb.12643 32291884

[B22] Menuz K. Larter N. K. Park J. Carlson J. R. (2014). An RNA-seq screen of the Drosophila antenna identifies a transporter necessary for ammonia detection. PLoS Genet. 10, e1004810. 10.1371/journal.pgen.1004810 25412082 PMC4238959

[B23] Mitchell R. F. Hughes D. T. Luetje C. W. Millar J. G. Soriano-Agatón F. Hanks L. M. (2012). Sequencing and characterizing odorant receptors of the cerambycid beetle *Megacyllene caryae* . Insect biochem. Mol. Biol. 42, 499–505. 10.1016/j.ibmb.2012.03.007 22504490 PMC3361640

[B24] Pelosi P. Iovinella I. Zhu J. Wang G. R. Dani F. R. (2018). Beyond chemoreception: Diverse tasks of soluble olfactory proteins in insects. Biol. Rev. Camb. Philos. Soc. 93, 184–200. 10.1111/brv.12339 28480618

[B25] Qi Q. L. Li D. X. Wang G. Y. Zang S. X. Cheng F. Z. (2008). Bionomics of the leaf beetle *Diorhabda tarsalis* . Chin. Bull. Entomol. 45, 975–978.

[B26] Sato K. Pellegrino M. Nakagawa T. Vosshall L. B. Touhara K. (2008). Insect olfactory receptors are heteromeric ligand-gated ion channels. Nature 452, 1002–1006. 10.1038/nature06850 18408712

[B27] Sharon D. Tilgner H. Grubert F. Snyder M. (2013). A single-molecule long-read survey of the human transcriptome. Nat. Biotechnol. 31, 1009–1014. 10.1038/nbt.2705 24108091 PMC4075632

[B28] Soffan A. Antony B. Abdelazim M. Shukla P. Witjaksono W. Aldosari S. A. (2016). Silencing the olfactory co-receptor RferOrco reduces the response to pheromones in the red palm weevil, *Rhynchophorus ferrugineus* . Plos One 11, e0162203. 10.1371/journal.pone.0162203 27606688 PMC5015987

[B29] Tatusov R. L. Galperin M. Y. Natale D. A. Koonin E. V. (2000). The COG database: A tool for genome-scale analysis of protein functions and evolution. Nucleic Acids Res. 28, 33–36. 10.1093/nar/28.1.33 10592175 PMC102395

[B30] The UniProt Consortium (2017). UniProt: The universal protein knowledgebase. Nucleic Acids Res. 45, D158–D169. 10.1093/nar/gkw1099 27899622 PMC5210571

[B31] Thomas S. Underwood J. G. Tseng E. Holloway A. K. (2014). Long-read sequencing of chicken transcripts and identification of new transcript isoforms. Plos One 9, e94650. 10.1371/journal.pone.0094650 24736250 PMC3988055

[B32] Wang G. R. Carey A. F. Carlson J. R. Zwiebel L. J. (2010). Molecular basis of odor coding in the malaria vector mosquito *Anopheles gambiae* . Proc. Natl. Acad. Sci. U. S. A. 107, 4418–4423. 10.1073/pnas.0913392107 20160092 PMC2840125

[B33] Wang Y. L. Chen Q. Zhao H. B. Ren B. Z. (2016). Identifcation and comparison of candidate olfactory genes in the olfactory and non-olfactory organs of elm pest *Ambrostoma quadriimpressum* (Coleoptera: Chrysomelidae) based on transcriptome analysis. Plos One 11, e0147144. 10.1371/journal.pone.0147144 26800515 PMC4723088

[B34] Wicher D. (2015). Olfactory signaling in insects. Prog. Mol. Biol. Transl. Sci. 130, 37–54. 10.1016/bs.pmbts.2014.11.002 25623336

[B35] Yan H. Opachaloemphan C. Mancini G. Yang H. Gallitto M. Mlejnek J. (2017). An engineered orco mutation produces aberrant social behavior and defective neural development in ants. Cell 170, 736–747. 10.1016/j.cell.2017.06.051 28802043 PMC5587193

[B36] Zhang B. Zhang W. Nie R. E. Li W. Z. Segraves K. A. Yang X. K. (2016a). Comparative transcriptome analysis of chemosensory genes in two sister leaf beetles provides insights into chemosensory speciation. Insect biochem. Mol. Biol. 79, 108–118. 10.1016/j.ibmb.2016.11.001 27836740

[B37] Zhang R. R. Gao G. Q. Chen H. (2016b). Silencing of the olfactory co-receptor gene in *Dendroctonus armandi* leads to EAG response declining to major host volatiles. Sci. Rep. 6, 23136. 10.1038/srep23136 26979566 PMC4793246

[B38] Zheng W. P. Liu Y. R. Zheng W. W. Xiao Y. L. Zhang H. Y. (2015). Influence of the silencing sex peptide receptor on *Bactrocera dorsalis* adults and offspring by feeding with ds-spr. J. Asia. Pac. Entomol. 18, 477–481. 10.1016/j.aspen.2015.05.004

[B39] Zhou Y. L. Zhu X. Q. Gu S. H. Cui H. H. Guo Y. Y. Zhou J. J. (2014). Silencing in *Apolygus lucorum* of the olfactory coreceptor Orco gene by RNA interference induces EAG response declining to two putative semiochemicals. J. Insect Physiol. 60, 31–39. 10.1016/j.jinsphys.2013.10.006 24216470

