# Supplementary figures and images for: Interference with orco gene expression affects host recognition in Diorhabda tarsalis
Source: Front Physiol. 2022 Dec 20;13:1069391. doi: 10.3389/fphys.2022.1069391 (PMC9808408; doi:10.3389/fphys.2022.1069391)

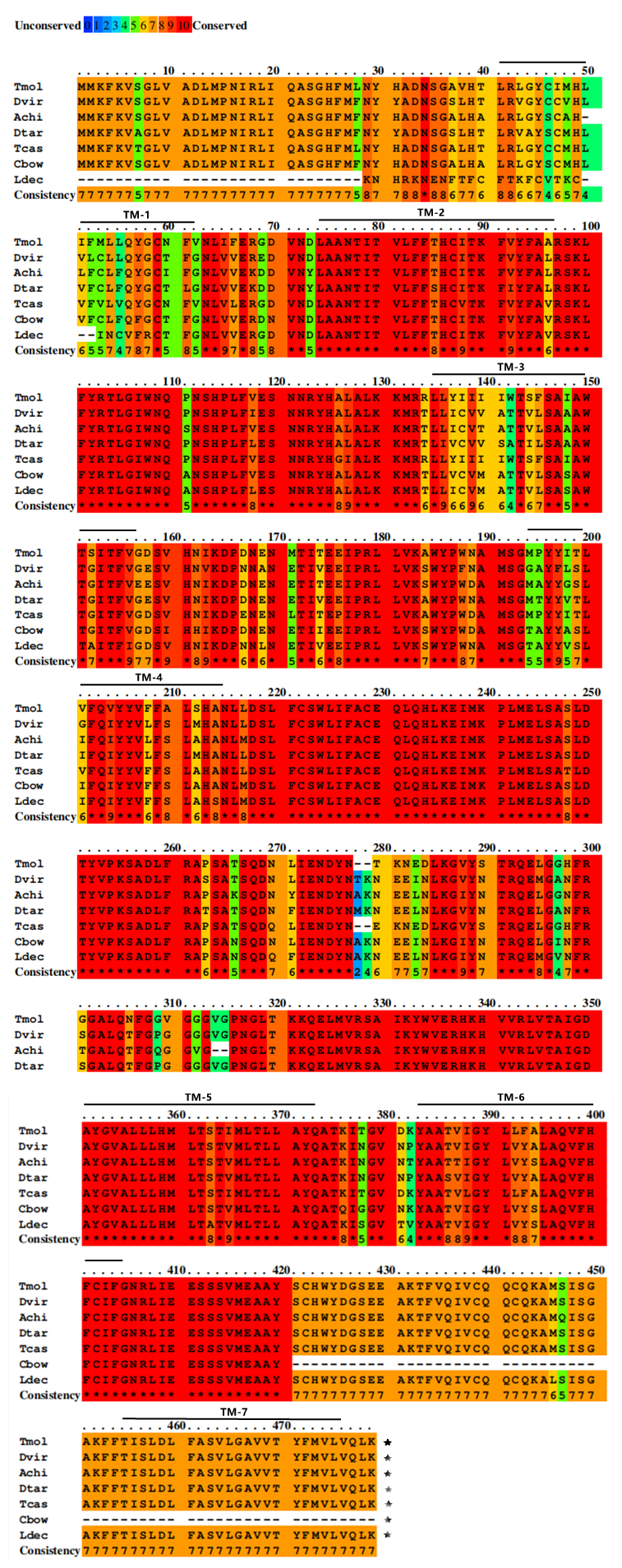

Supplement: Supplementary file 1 [file Image3.JPEG]

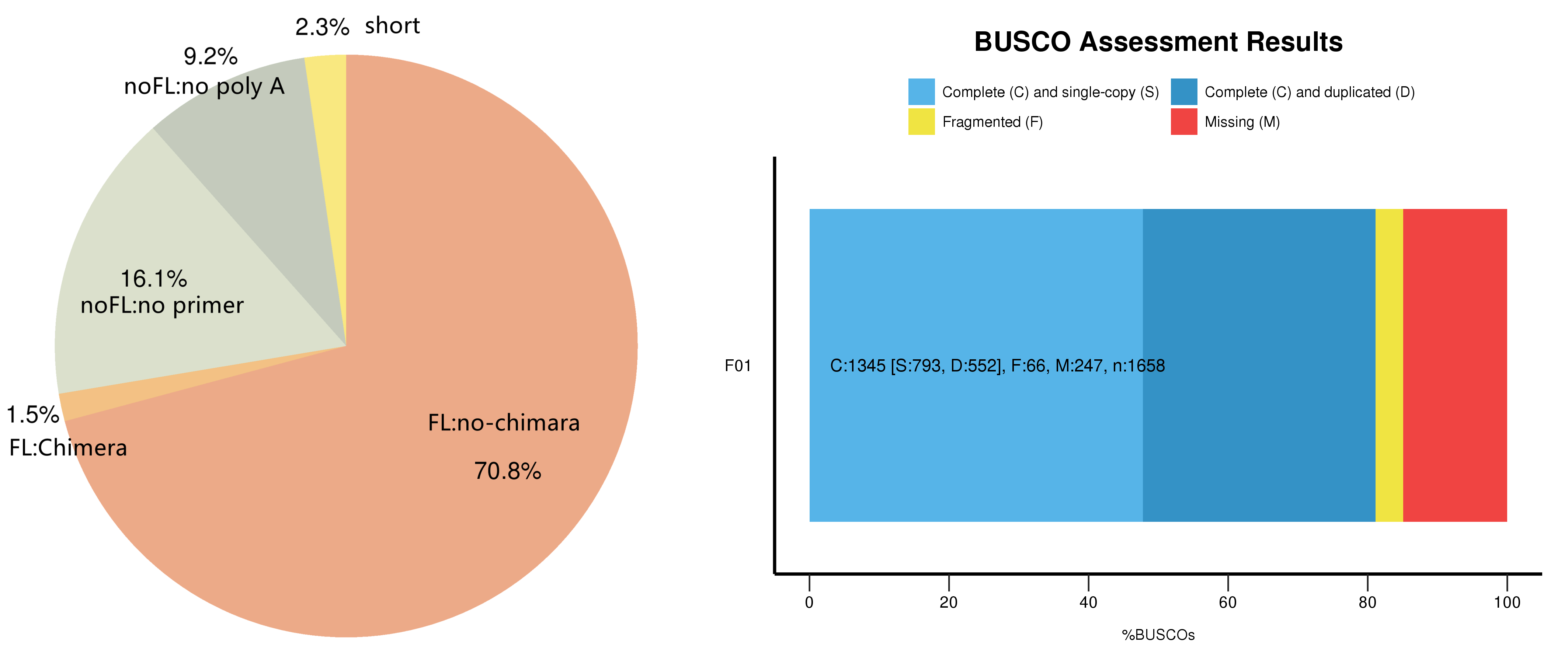

Supplement: Supplementary file 3 [file Image1.JPEG]

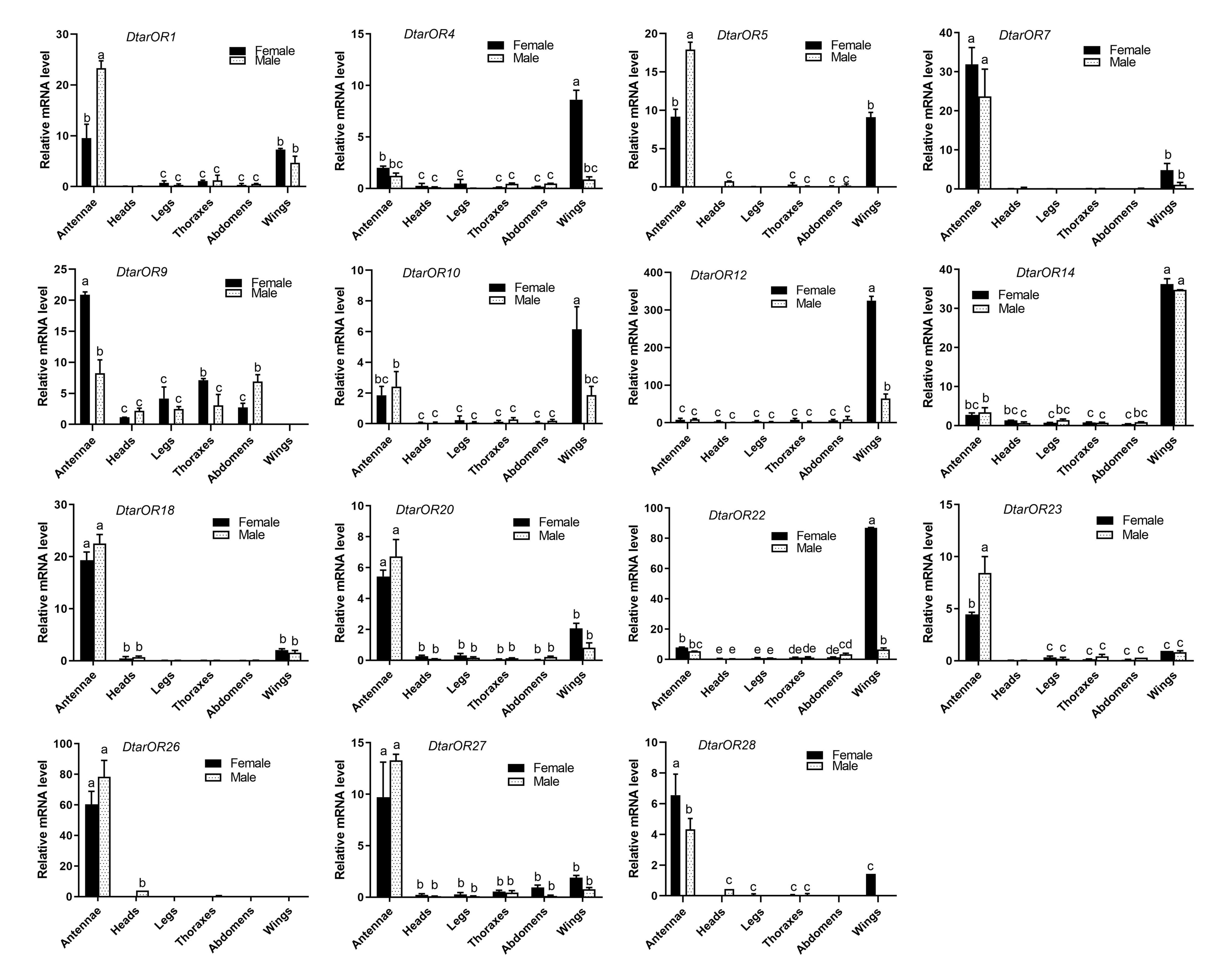

Supplement: Supplementary file 4 [file Image4.JPEG]

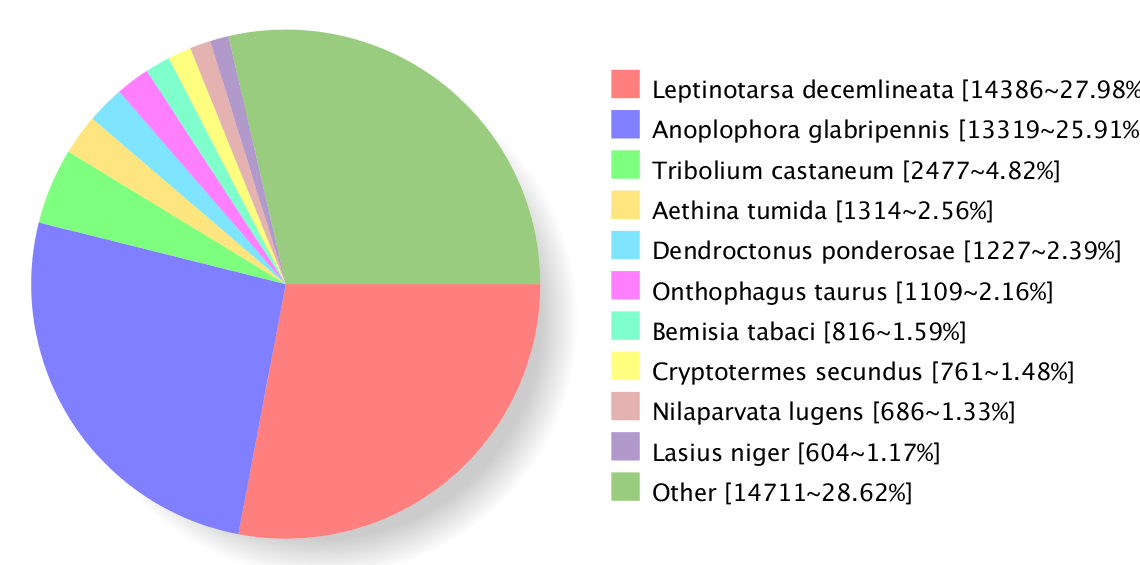

Supplement: Supplementary file 5 [file Image2.TIF]

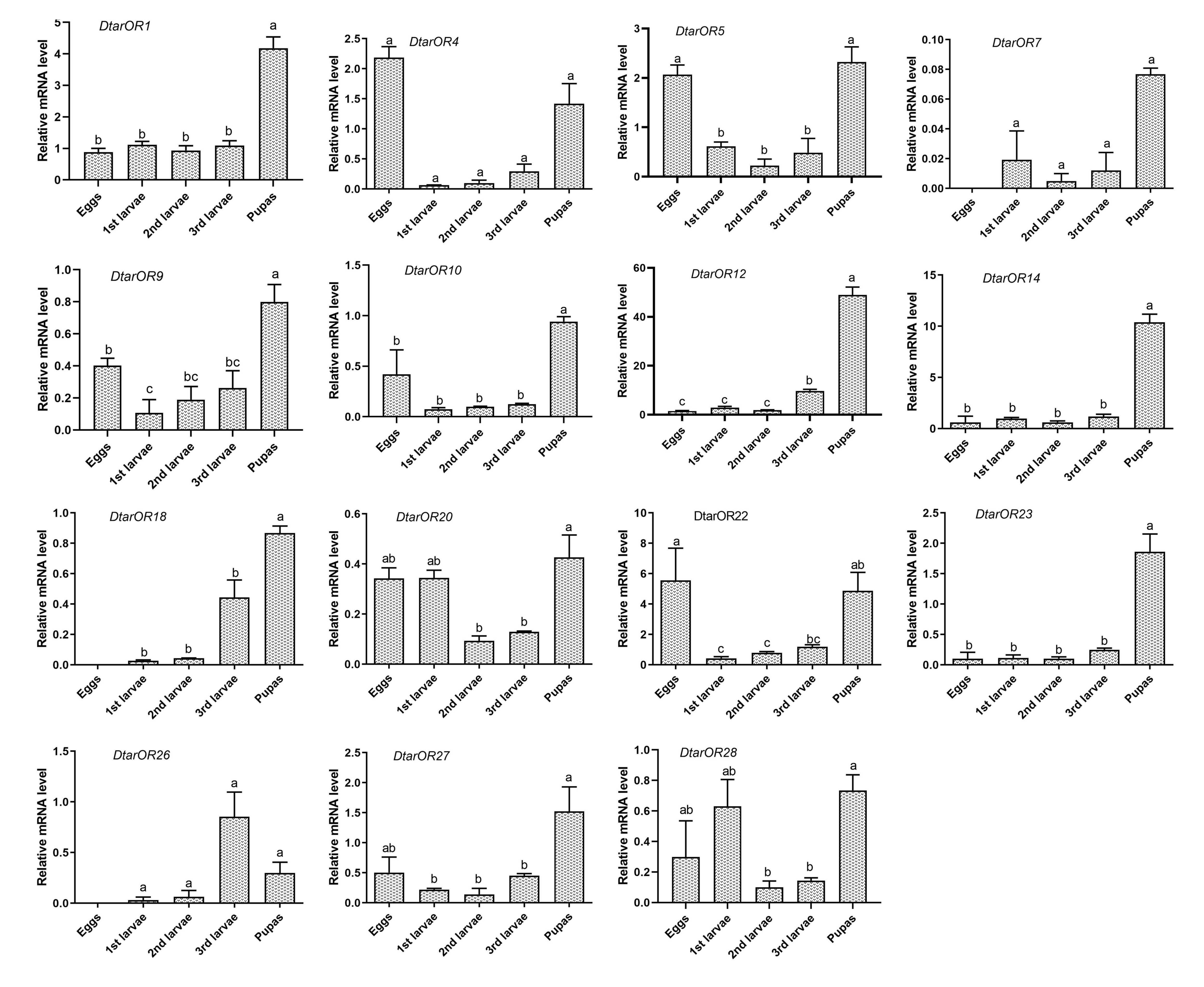

Supplement: Supplementary file 6 [file Image5.JPEG]
