# Supplementary material for: Interference with orco gene expression affects host recognition in Diorhabda tarsalis
Source: Front Physiol. 2022 Dec 20;13:1069391. doi: 10.3389/fphys.2022.1069391 (PMC9808408; doi:10.3389/fphys.2022.1069391)
Supplement: Supplementary file 2 [file Table1.pdf]

Table S1 RT-qPCR quantitative primers of partial ORs.

Gene	Forward primer	Reverse primer
DtarORco	GCACGCAAATCTATTGGACAG	AGCACTCTTCGGAACGTAAG
DtarOR1	GAGCAATTGATTCCACCGGC	TCGCACAATGAGTAACTGCT
DtarOR4	TGTTTCGTCAACTTCCCGCT	TTCGCTGCGGGATCTCATAC
DtarOR5	ATCACGCCGATATTGTAGCAA	ATCCAATTCGAACCAATCACTTTC
DtarOR7	TGTGTACAGGATGGTTTTGGG	TCCTCACTAAAATCATACCACGG
DtarOR9	ACGTGGCTTCCCTTTGACAT	TTTCGAACACATGCCAAGCC
DtarOR10	TTACCGATGGCAACTTGGCT	TCATTTCAGCAACACTGGACT
DtarOR12	AATTCGCCCCACTCCAAG	CCATGTGCCTTGAAACTGTG
DtarOR14	AACCCCTTTGATTGGCTACA	ACATGACACCGACGCTTAATG
DtarOR18	GCTCACGCAACAATAACG	ATTCCAACGATGCTGTAAGA
DtarOR20	TGTGATTGGATGAGTGGCAG	AACAGCTACGATGGTACTCAAC
DtarOR22	ACGACATTTCAGAGTGGTTGGT	TCACCAAACCAGGTGTAGCA
DtarOR23	GACGAAGAAGAAGAGAAGATAC	CAGTGCGAACCAGAGATAA
DtarOR26	TGTTGGATGGCTTTCGGG	CATGGTAAGTCGTAAACTGCG
DtarOR27	AACATTTTCGGTATTTGGACGG	ACATTGTCTCTTGCTTTGTACATG
DtarOR28	TGGTCGTTTACTCGACGCTC	ATAAGATCGCATTGTGCGCC
Tubulin	TCTGCCACTTTCATCGGTAAC	CCATTTCATCCATACCTTCTCCG

Table S2 PacBio libraries and sequencing results.

cDNA size	Reads of insert	Read bases of insert	Mean read length of inserts	Mean read quality of inserts	Mean number of passes
1-6K	632,484	1,517,111,365	2,398	0.95	14
All	632,484	1,517,111,365	2,398	0.95	14

Tables S3 Summary of sequencing reads after filtering.

cDNA Size	Reads of Insert	Number of filtered short reads	Number of nFL reads	Number of FL reads	Number of FL chimeric reads	Number of FL non-chimeric reads	Average FL non-chimeric read length
1-6K	632,484	14,575	160,159	457,750	9,667	448,083	2,203
All	632,484	14,575	160,159	457,750	9,667	448,083	2,203

Table S4 Summary of functional annotation results.

Annotated databases	number of isoforms
COG	13,877
GO	13,372
KEGG	19,189
KOG	27,408
Pfam	29,758
Swiss-Prot	24,907
eggnog	45,669
Nr	51,424
All	52,436
